# The burden of burnout: Understanding its prevalence and organizational drivers in medical physics

**DOI:** 10.1002/acm2.70121

**Published:** 2025-05-20

**Authors:** Deborah Schofield, C. Lynn Chevalier, Laurence Court, William Harmsen, Akiva Turner

**Affiliations:** ^1^ Department of Health Science Nova Southeastern University Fort Lauderdale Florida USA; ^2^ Department of Radiation Physics University of Texas MD Anderson Cancer Center Houston Texas USA; ^3^ Department of Quantitative Health Sciences Mayo Clinic Rochester Minnesota USA

**Keywords:** burnout, medical physicist, quality, safety

## Abstract

**Background:**

Burnout is a work‐related syndrome characterized by increased levels of emotional exhaustion (EE) and depersonalization (DP) along with decreased levels of personal achievement. In the healthcare setting, higher burnout levels have been associated with negative impacts on personnel, an increased risk of errors, and a decrease in the quality of delivered care.

**Purpose:**

The purpose of this study was to assess the prevalence of burnout among medical physicists working in the United States. Additionally, the impact of personal and organizational features on burnout risk was examined.

**Methods:**

The anonymous survey was distributed to 1962 full members of the American Association of Physicists in Medicine. The survey consisted of seven demographic questions, the validated Maslach Burnout Inventory (MBI), and an organizational assessment tool. Burnout risk was evaluated using two different scoring methods. Inferential statistics were employed to examine the relationship between burnout and personal features, such as practiced sub‐specialty, and organizational features, including the respondent's assigned facility safety score.

**Results:**

A total of 337 responses were received, and 59.9% of medical physicist participants scored high on at least one burnout domain. A statistically significant association was found between the EE and DP burnout domains and personal factors, including working as a therapy medical physicist, working longer hours, and a moderate or significant impact on work‐related feelings due to the COVID‐19 pandemic. A statistically significant relationship was identified between all three burnout domains and the respondent's assigned facility safety score. Amongst therapy physicists, an inverse relationship was observed between all three burnout domains and both the teamwork and staffing constructs, as well as the open communication and punitive concerns construct.

**Conclusions:**

Medical physicists in the United States are experiencing significant levels of burnout. Importantly, this study identified a link between quantitative burnout scores and facility safety, stressing the importance of addressing burnout.

## 1 INTRODUCTION

Since burnout first emerged in the scientific literature in the mid 1970s, there has been intense interest in the topic, including a near exponential growth of publications on burnout in health care workers.^1^ Although the term is often used colloquially as a synonym of fatigue, the prevailing formal definition identifies burnout as a work‐related condition comprised of three dimensions, including emotional exhaustion (EE), cynicism or depersonalization (DP), and feelings of reduced personal achievement (PA).^2^ EE is expressed by emotional depletion, often in response to a chronic condition of high workload with low resources/time. Depersonalization manifests as a dehumanizing response to those patients or clients the individual is tasked with serving, while decreased feelings of adequacy and competence are associated with the third dimension of reduced PA. The World Health Organization (WHO) first included burnout in the International Classification of Diseases (ICD‐10): Classification of Mental and Behavioral Disorders,^3^ where it was identified as a condition that can impact health status or cause an individual to seek professional services. In ICD‐11,^4^ WHO provided an expanded burnout definition, giving further legitimacy to the syndrome. Although EE is the best‐known and researched of the three burnout dimensions, it is important to acknowledge that unfavorable changes in the PA and DP dimensions suggest that burnout negatively alters one's relationship with themselves and others.

In addition to the detrimental consequences on the individual experiencing burnout, there can be significant negative implications for patients who receive care from these professionals, including a direct relationship between burnout and errors. A longitudinal study of internal medicine residents (*n* = 184) at a single institution found that each 1‐point increase on the validated, Maslach Burnout Inventory's (MBI) EE and DP scales were associated with a 7% and 10% respective increase in the odds of self‐reporting a major medical error in the following quarter.^5^ Similarly, a cross‐sectional survey of American surgeons (*n* = 7905) found that each 1‐point increase on the MBI's DP and EE scales, respectively, were associated with an 11% and 5% increase in the odds of having a self‐reported error in the prior 3‐month period.^6^ A direct relationship between burnout and patient satisfaction with the quality of received care has also been identified. Researchers utilizing the MBI (*n* = 820 nurses) and the La Monica‐Oberst Patient Satisfaction Scale (*n* = 621 patients) at 40 inpatient units across 20 hospitals found that patients on units with above‐average levels of EE among the nursing staff were half as likely to be highly satisfied with the care they received.^7^

Staff burnout can have a significant negative impact on the work environment. In the United Kingdom, a study found that 42% of radiation oncology staff experienced presenteeism, a condition where employees are physically at work but feel unable to perform their duties.^8^ Additionally, 77% of responding radiology practice managers (*n* = 367), representing 30% of all practicing radiologists in the United States, reported that burnout was either a very significant (55%) or significant (22%) problem in their practice.^9^

The link between organizational features and burnout risk has also been explored. A systematic review found that positive organizational changes, like adopting team‐based approaches to patient care, were directly associated with reduced burnout among physicians.^10^ Researchers also identified that the leadership score of the immediate supervisor was inversely related to burnout risk and directly related to overall employee satisfaction with the organization.^11^

Although numerous studies have been conducted to evaluate burnout in medical professionals, data on its prevalence in medical physicists is sparse despite evidence that therapy medical physicists have the highest scores for workload and mental demand among radiation oncology staff.^12^ In one study investigating the need for social support among medical physicists, 32.8% of 1,001 respondents self‐reported experiencing frequent or constant burnout.^13^ A European study focused exclusively on medical physicists investigated burnout and its relationship with other measured personal factors.^14^ Among the 308 participants, 30% scored high in burnout, and a positive correlation was found between the risk of experiencing burnout and alexithymia, a condition where an individual has difficulty identifying and expressing emotions.

Medical physicists play a clear and crucial role in maintaining the quality of care within their purview. Despite existing literature that highlights a connection between burnout and medical errors, there is currently a limited understanding of the prevalence of burnout among medical physicists in the United States. Further, the relationship between the syndrome and personal and organizational features among this cohort has been unexplored. This is the first study to utilize a validated survey tool to assess burnout and evaluate its correlation with personal and organizational features among medical physicists in the United States.

## 2 MATERIALS AND METHODS

The Executive Committee of the American Association of Physicists in Medicine (AAPM) gave approval to survey the membership for this research. The Institutional Review Board at Nova Southeastern University reviewed and approved this study (IRB# 2020–407).

### 2.1 Survey instrument

The survey instrument used in this study consisted of three sections, including a demographics component, the Maslach Burnout Inventory‐Human Services Survey (MBI‐HSS), and an organizational survey. The complete instrument was built on an online survey platform (SurveyMonkey Inc., San Mateo, California). A small test group verified the clarity of instructions and accurate transcription of each instrument. Additionally, the test group confirmed that the web‐based survey platform effectively prevented multiple submissions from the same individual and from those not on the invitation list.

#### 2.1.1 Demographics

The demographics section consisted of seven questions, including years in practice, work setting, group size, and subspecialty. A single demographic question was included to determine the impact of the COVID‐19 global pandemic on work‐related feelings. Since the relationship between burnout risk and geography and cultural background is not fully understood, participants were asked to identify the percentage of their adult life lived in North America.

#### 2.1.2 MBI

The MBI is a validated, 22‐item survey with 9, 5, and 8 questions used to establish the EE, DP, and PA burnout domains, respectively. Respondents utilized a 7‐point Likert scale, with options ranging from never to every day, to indicate the frequency they experience each emotion. The MBI was chosen for this work because the instrument is considered the “gold standard” for burnout research and has been used in more than 80% of all burnout publications.^15^ In one meta‐analysis of 84 articles where the MBI was utilized, the mean Cronbach's alpha coefficient, which measures an instrument's internal validity, was found to range between 0.7 and 0.8 for each of the three burnout domains.^16^ Copyright limitations prevent a full reproduction of the MBI, but approved example questions are provided in Table 1. The survey instructions for this work defined the term “recipient” as any individual to whom the participant provides professional service and expertise, including patients and co‐workers. The appropriate licenses to utilize the MBI for this work were purchased from Mind Garden (Menlo Park, California).

**TABLE 1:** Approved MBI sample questions for the three burnout domains.

Burnout Domain	Sample Question
EE	I feel emotionally drained from my work
PA	I have accomplished many worthwhile things in this job
DP	I don't really care what happens to some recipients

*Note*: A 7‐point Likert‐scale is used to identify the frequency with which respondents experience each emotion.

Abbreviations: DP, depersonalization; EE, Emotional Exhaustion; MBI, Maslach Burnout Inventory; PA, personal achievement.

#### 2.1.3 Organizational survey tool

The organizational survey instrument utilized in this work was a slightly modified version^17^ of the validated, open‐access Survey on Patient Safety Culture version 1 (SOPS 1.0) designed by the Agency for Healthcare Research and Quality.^18^ Modifications to the SOPS v1.0 survey included the removal of questions related to shift and inter‐unit hand‐offs, which are common in hospital settings but not typically relevant to medical physics. Additionally, questions gauging participants' attitudes towards error reporting were added. While reliability tests were not reported on the modified survey, the original SOPS 1.0 survey was found to have a mean Cronbach's alpha coefficient of 0.77 (range 0.62 to 0.85) across all evaluated themes.^19^

The organizational survey portion consisted of 67 questions on five main themes, including a) teamwork and staffing, b) responsibility and efficacy, c) open communication and punitive concerns, d) feedback, and e) patient safety perceptions. Except for four open‐ended questions, all items in this instrument utilized multiple‐choice, yes/no, or Likert scale response formats.

### 2.2 Data collection

The AAPM provided the names and email addresses of a random sampling of 50% of the organization's full members residing in the United States (*n* = 1,962). Full membership in the organization excludes, for instance, students (undergraduate and graduate students), residents, and postdoctoral fellows. The initial invitation to participate in this study was sent on November 10, 2020. To avoid biasing responses, the term “burnout” was intentionally avoided in recruitment materials. Potential participants were advised that the research was to assess job‐related attitudes among medical physicists. Anonymity was assured, and affirmative consent was required for participants to proceed to data collection. Data collection was open for a period of 4 weeks, with the survey closing on December 9, 2020. Reminder emails were sent to those who had not completed the survey approximately 2 weeks and 24 h prior to the close of data collection.

### 2.3 Data analysis

Raw data was downloaded and cleaned prior to analysis. Responses from individuals who did not meet the inclusion criteria and responses from individuals who did not move past the demographics section of the survey tool were removed. All data analysis was completed with the Statistical Package for Social Sciences (SPSS), version 27 (IBM SPSS, Armonk, New York), SAS/STAT, version 9.4 (SAS Institute Inc., Cary, North Carolina) or R, version 4.2.2 (The R Foundation for Statistical Computing, http://www.r‐project.org/).

In addition to descriptive statistics, the internal consistency of both the MBI and the organizational instrument was assessed by calculating the Cronbach's alpha coefficient, with a score of 0.7 or higher deemed acceptable. An independent samples t‐test or ANOVA was conducted to examine significant differences between groups based on medical physics sub‐specialty, facility setting, respondent assigned facility safety grade, and hours worked per week. The Tukey‐Kramer pairwise comparison post‐hoc test was conducted when results from the ANOVA indicated statistically significant differences between comparison groups. When appropriate, effect sizes were also computed using Cohen's d. The Pearson‐product moment correlation was used to assess the relationship among therapy physicists between EE and two organizational constructs: teamwork and staffing, and open communication and punitive concerns. Finally, regression analysis was conducted to examine the relationships between the COVID‐19 impact, facility safety grade, teamwork and staffing, and open communication and punitive concerns with each of the burnout domains.

## 3 RESULTS

### 3.1 Participants

1962 full members of the AAPM with a United States‐based practice location were contacted for inclusion in this study. Postdoctoral fellows and individuals in training, including students and residents, were excluded. Since a key component of the research was to evaluate the correlation between burnout and clinical organizational features, participants working for a consulting group providing professional services to multiple institutions at the same time and individuals working for vendors were excluded.

There were 34 (1.7%) invalid email addresses, and 63 individuals (3.2%) opted out of the survey and further email reminders. An additional 728 (37.2%) invitations were never opened. With an average survey completion time of just under 16 min, 387 responses were received, representing a 20.1% response rate of deliverable invitations.

Of the 387 responses, 40 participants did not proceed past the demographics section and were excluded from data analysis. An additional 10 participants were removed for failing to identify their practice location (2), for practicing outside of the United States (1), or for indicating that they worked for a vendor (7). The clean data set used for analysis had a total of 337 responses.

### 3.2 Demographics

The demographic characteristics of respondents included in the clean data set are summarized in Table 2. 17.5% of respondents indicated working in a solo practice while those working in small groups (2‐3 physicists), medium groups (4‐10 physicists), and large groups (≥11 physicists) comprised 29.1%, 31.1%, and 22.3% of respondents respectively. There was a nearly equivalent representation of work environments with 33.5% of respondents working in a community‐based practice, 36.2% in an academic‐affiliate practice, and 30.3% of respondents working in other settings, including government, free‐standing, and consulting positions.

**TABLE 2:** Summary of the demographic characteristics of respondents.

Practice type	%	*n*	Physicists in practice	%	*n*
Academic‐affiliated	36.2	122	1	17.5	59
Community	33.5	113	2–3	29.1	98
Government	3.0	10	4–5	11.9	40
Free‐standing	11.6	39	6–10	19.2	65
Consulting	13.0	44	11–20	10.4	35
Other	2.7	9	>20	11.9	40

Primary specialization^a^	%	*n*	Impact of COVID‐19 on job‐related feelings	%	*n*
Therapy	72.3	243	None	20.2	68
Diagnostic	22.0	74	Very mild	18.7	63
Health physics/RSO	1.8	6	Mild	25.8	87
Nuclear medicine	3.0	10	Moderate	25.8	87
Other	0.9	3	Significant	9.5	32

Years of post‐graduate experience^a^	%	*n*	Percentage of Life spent in North America^a^	%	*n*
0–2	3.0	10	<25%	1.5	5
3.5	4.7	16	25%–50%	3.3	11
6–10	1.2	4	51%–75%	12.8	43
11–15	1.2	4	>75%	82.4	277
16–20	15.2	51			
21+	74.7	251			

^a^ Three demographics questions have only 336 responses due to a missing response from three separate participants.

Therapy physicists and diagnostic physicists comprised 72.1% and 22% of respondents, respectively. Although there was no minimum level of experience required to participate in this study, 89.6% of respondents had 16 or more years of post‐graduate work experience, and almost all participants (95%) spent most of their adult life in North America. Finally, nearly two‐thirds (64.7%) of respondents indicated that the COVID‐19 pandemic had no to only a mild impact on their job‐related feelings. The remaining participants indicated that the pandemic had a moderate (25.8%) or significant (9.5%) impact on how they felt about their job.

### 3.3 MBI

**FIGURE 1:**
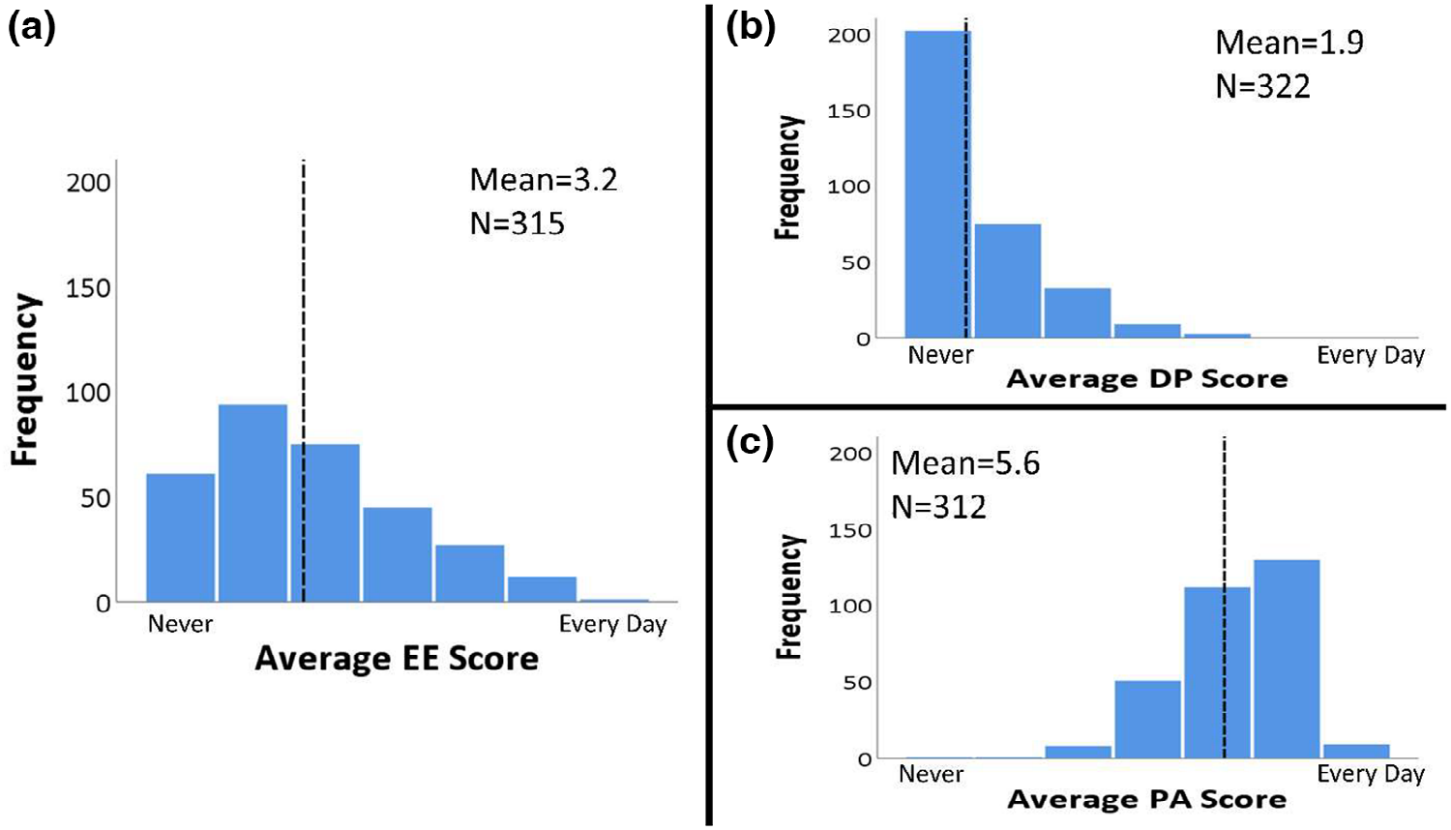
Histograms of the average scores for all participants on the EE (a), DP (b), and PA (c) domains. The mean domain score across all participants is denoted by the black line. A higher risk of burnout is indicated by higher scores on the EE and DP domains and a lower score on the PA domain. DP, depersonalization; EE, emotional exhaustion; PA, personal achievement.

The internal consistency of the three MBI dimensions was calculated prior to data analysis. The Cronbach's alpha coefficients were 0.93, 0.70, and 0.75 for the EE, DP, and PA constructs, respectively. Average scores for each burnout domain were calculated across all participants who answered every question for the respective construct. The response distributions for each domain are summarized in Figure 1. Higher scores on the EE and DP domains and a lower score on the PA domain indicate a higher level of burnout burden. On average, respondents experience feelings of PA a few times each week, while EE is experienced a few times per month, and DP is experienced less than once per month.

Responses were analyzed using summed domain scores and the historical cut score method (Table 3). The MBI creators initially developed the cut score methodology by dividing the scores of a large reference population into three equal groupings.^20^ Although the cut score methodology is no longer the preferred method for categorizing burnout risk, the results are presented for context and ease of comparison to the voluminous published burnout data that used this methodology. On the EE domain, 50.8% of respondents scored in the high‐risk category, with an additional 32.4% scoring in the moderate risk range. For the DP and PA domains, 20.5% and 3.2% scored in the high‐risk category, respectively.

**TABLE 3:** Burnout classification of risk using the historical cut score technique.

Burnout Risk	Cut Score	% of Respondents	n
*EE*			
**High**	27+	**50.8**	160
Moderate	17–26	32.4	102
Low	0–16	16.8	53
*DP*			
**High**	13+	**20.5**	66
Moderate	7–12	47.5	153
Low	0–6	32	103
*PA*			
High	39+	83.3	260
Moderate	32–38	13.5	42
**Low**	0–31	**3.2**	10

*Note*: Higher burnout risk (bolded) is indicated by higher scores on the EE and DP domains and a lower score on the PA domain.

Abbreviation: DP, depersonalization; EE, emotional exhaustion; PA, personal achievement.

Responses were also analyzed by calculating the critical boundary (*z*‐score threshold), which is the current, preferred method for evaluating MBI scores. This scoring methodology was developed by the creators of the MBI using latent profile analysis.^21^ This approach means that scoring is based on the mean and standard deviation (SD) of the sampled population for each of the three domains, rather than the historic arbitrary cut score technique. The equations to calculate critical boundaries and results are summarized in Table 4. A total of 30.1% of participants exceeded the critical boundary on the EE domain. With a mean score of 5.0, individuals in this group experience EE a few times each week compared to the sample average of a few times per month. Although only 12.4% of respondents surpassed the critical DP threshold, the mean score for this group was double that of the overall sample, indicating that they experience DP almost weekly. Additionally, 46.5% of participants exceeded the PA critical boundary. However, with a mean score of 4.9, the group still experiences feelings of PA at least weekly.

**TABLE 4:** Burnout risk using the critical (*z*‐score) thresholds.

	EE	DP	PA
*Z Score equation*	Mean + (SD*0.5)	Mean + (SD*1.25)	Mean + (SD*0.1)
Mean (total sample)	3.2	1.9	5.6
SD	1.4	0.9	0.9
*Z*‐Score threshold	3.9	3.0	5.7
Respondents at risk	30.1% (*n* = 95)	12.4% (*n* = 40)	46.5% (*n* = 145)
Mean score (at risk)	5.0	3.8	4.9

*Note*: The mean domain score was calculated for the subset of participants who exceeded the critical boundary.

Abbreviations: DP, depersonalization; EE, emotional exhaustion; PA, personal achievement; SD, standard deviation.

**FIGURE 2:**
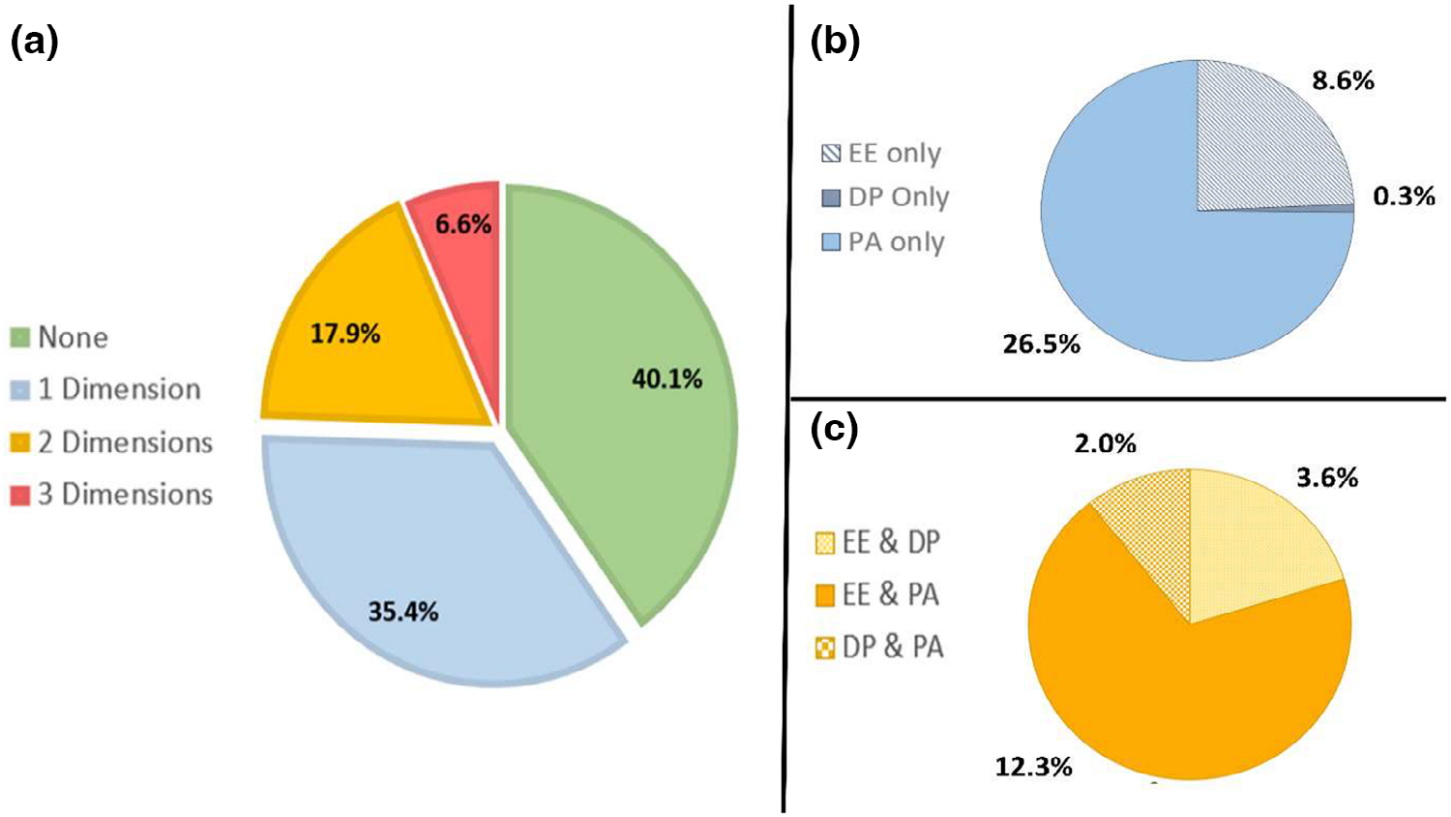
(a) Burnout risk among medical physicists utilizing the critical threshold scoring methodology. Among the respondents, 40.1% scored below the critical threshold in all three domains, suggesting they are engaged employees. 6.6% scored above the critical threshold on all three domains, suggesting burnout. The remaining 53.3% fell into an intermediate category, with 35.4% and 17.9% exceeding the critical threshold on 1 and 2 domains, respectively. Without intervention, these individuals are at risk of progressing to full burnout. (b) Distribution of participants who exceeded the critical threshold on only one domain. (c) Distribution of participants who exceeded the critical threshold on two domains.

Utilizing the critical boundary scores, a composite analysis was performed (Figure 2). Of the respondents, 40.1% scored below the critical threshold across all three domains and can be classified as engaged professionals. The remaining 59.9% exceeded the critical threshold on at least one domain. Specifically, 6.6% of participants exceeded the critical threshold across all three domains, suggesting they are experiencing full burnout, while the remaining 53.3% fell into an intermediate category, indicating they are at risk of developing full burnout without intervention.

### 3.4 Work environment and burnout risk

The relationship between each of the burnout domains and the work environment were evaluated for all respondents.

#### 3.4.1 Sub‐Specialty

Differences in responses across the three burnout domains were analyzed based on sub‐specialty. Due to the small number of respondents in some sub‐specialties, those who reported working in diagnostic, health, or nuclear medicine physics were combined into a “non‐therapy” grouping and compared to those practicing therapy physics. Participants who selected “other” as their sub‐specialty were excluded from this analysis. An independent samples *t*‐test and Cohen's d were utilized to assess statistical significance and effect size, respectively. Therapy physicists showed significantly higher levels of EE (3.28 vs. 2.90, *p* = 0.027, *d* = 0.27) and DP (1.96 vs. 0.93, *p* < 0.001, *d* = 0.42) compared to non‐therapy respondents. However, no significant difference was found between the two groups on the PA domain (5.65 vs. 5.63, *p* = 0.86).

#### 3.4.2 Facility setting

Differences in responses across the three burnout domains were evaluated based on the work environment for all respondents. Those who reported working in community‐based, government, or free‐standing facilities were combined in a “non‐academic” grouping and compared to respondents working in an academic environment. Respondents working for a consulting group or “other” setting were excluded from this analysis. An independent samples *t*‐test was conducted, and no statistically significant differences were found between the two groups.

The analysis was repeated separately for therapy and non‐therapy physicists. A significant difference was observed only for the EE domain among therapy physicists. Specifically, therapy physicists employed in an academic setting reported significantly higher EE (3.57 ± 1.40) compared to those employed in non‐academic settings (3.16 ± 1.47, p = 0.049, d = 0.28).

#### 3.4.3 Number of physicists

Using Tukey–Kramer pairwise comparisons, solo physicists demonstrated significantly lower EE (2.46 vs. 3.34, *p* < 0.0001) and DP scores (1.6 vs. 1.95, *p* = 0.0093) than respondents working in an environment with 2 or more physicists. The number of facility physicists was not statistically significant on the PA domain (5.7 vs. 5.63, *p* = 0.5808).

#### 3.4.4 COVID‐19

**FIGURE 3:**
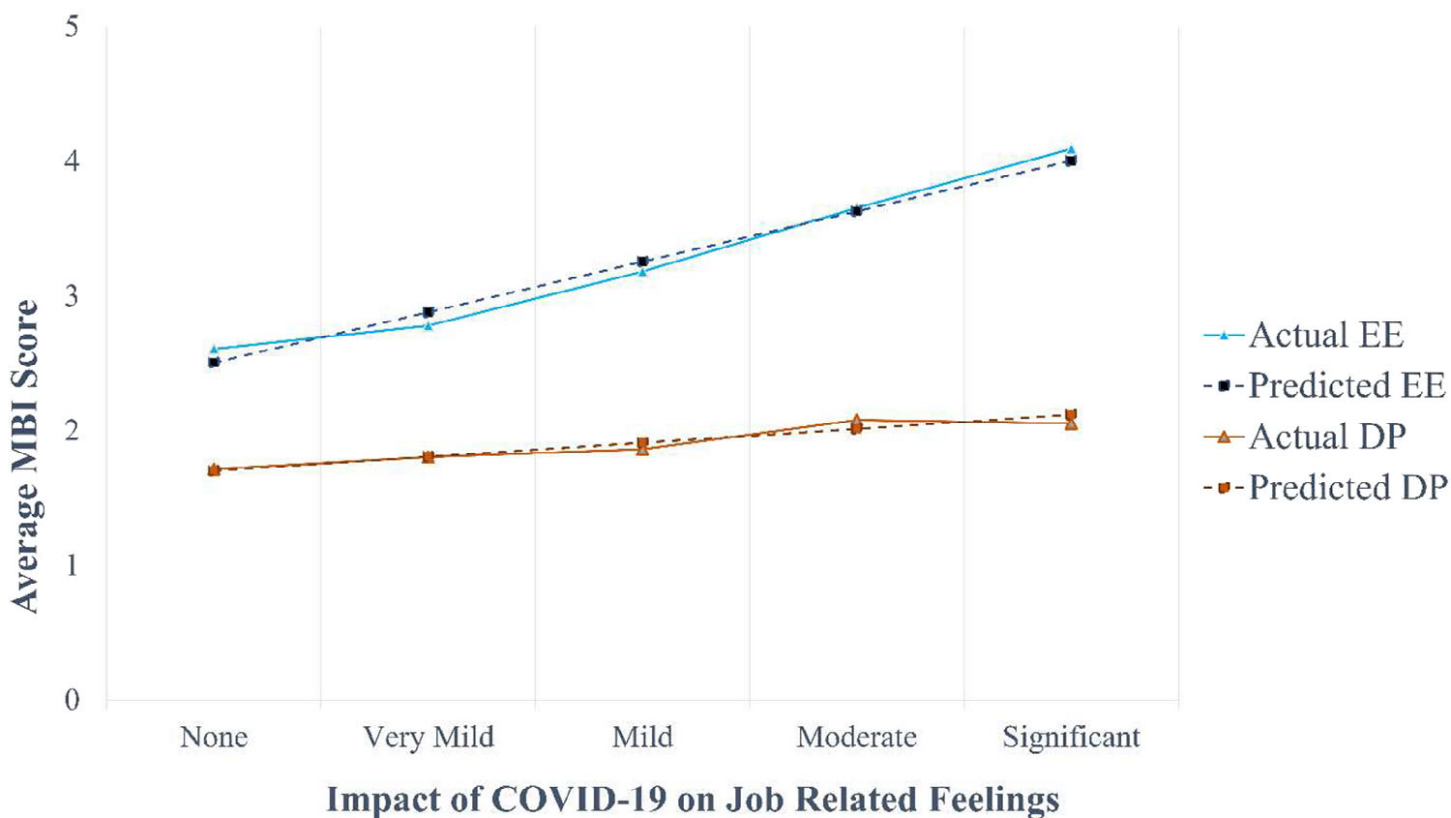
Average EE and DP scores as a function of the impact of COVID‐19 on respondents’ job‐related feelings. There is excellent agreement between the average domain scores and the scores predicted using a linear regression model. No statistically significant relationship between the COVID‐19 impact score and the PA burnout domain was observed. DP, depersonalization; EE, emotional exhaustion; PA, personal achievement.

Respondents utilized a 5‐point scale to indicate the impact of COVID‐19 on their job‐related emotions, ranging from none to significant. Tukey–Kramer pairwise comparisons revealed that respondents who reported no, very mild, or mild impact from Covid‐19 on their job‐related feelings had significantly lower EE (2.88 vs. 3.77, *p* < 0.0001) and DP (1.8 vs. 2.07, *p* = 0.0097) compared to those who reported a moderate or significant impact. However, COVID‐19 did not have a statistically significant effect on the PA domain (5.63 vs. 5.67, *p* = 0.697). Using linear regression (Figure 3), each incremental increase in the Covid‐19 impact score (e.g., from mild to moderate), was associated with a 0.37‐point increase on the EE Likert scale (*p* ≤ 0.0001) and a 0.1‐point increase on the DP Likert scale (*p* = 0.009).

#### 3.4.5 Hours worked per week

Participants were asked to report their average weekly work hours using a six‐category ordinal scale. Of the 282 responses, the majority (74.1%) reported working between 40 and 59 h per week, while 14.9% worked 39 h or less each week. The remaining 11% worked between 60 and 99 h each week. A statistically significant difference between these three groups was found on both the EE domain (*p* < 0.0001) and the DP domain (*p* = 0.0234), with EE and DP scores increasing with increasing work hours. However, no statistically significant relationship was found between work hours and the PA domain (*p* = 0.6927). Of note, among those working between 60 and 99 h per week, 77.4% were therapy physicists, 19.4% were diagnostic physicists, and 3.2% chose the “other” category. Additionally, within this group, 41.9% reported working in an academic facility, 41.9% in a community hospital, 6.5% in a free‐standing facility, and 9.7% as consultants.

#### 3.4.6 Department safety grade

**FIGURE 4:**
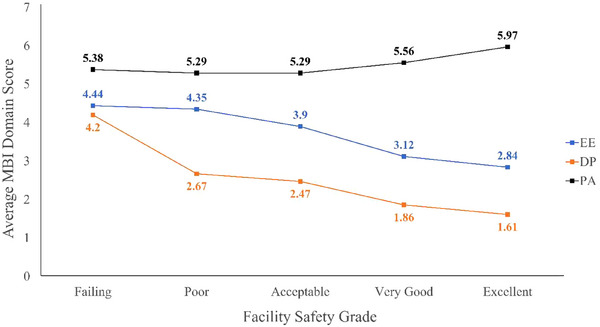
Average burnout domain scores as a function of the respondent's assigned facility safety grade. As the facility safety grade increased, EE and DP decreased while PA increased. Lower burnout risk is indicated by lower scores on the EE and DP domains and a higher score on the PA domain. DP, depersonalization; EE, emotional exhaustion; PA, personal achievement.

Participants were asked to provide an overall patient safety grade for their department using a 5‐point scale ranging from failing to excellent. Of the 281 responses, 31.3% rated their facility as excellent and 53.4% as very good. The remaining 15.3% of respondents gave their department a patient safety score of acceptable, poor, or failing. An ANOVA analysis showed a statistically significant relationship between the department's safety grade and all 3 burnout domains. As facility safety grades decreased, EE and DP scores increased, and PA scores decreased, indicating higher levels of burnout (Figure 4). Using linear regression, each one‐level decrease in facility safety grade (e.g., from excellent to very good) was associated with a 0.48‐point increase on the EE Likert scale (*n* = 265, *p* < 0.0001), a 0.43‐point increase on the DP Likert scale (*n* = 273, *p* < 0.0001), and a 0.3‐point decrease on the PA Likert score (*n* = 260, *p* < 0.0001).

#### 3.4.7 Organizational features and burnout risk in therapy physicists

The relationship between burnout and two organizational features was evaluated. The survey questions attributed to the teamwork and staffing and open communication and punitive concern constructs are provided in Table 5. Prior to analysis, the responses to negatively worded questions were reverse‐coded, and a sum score was generated for each organizational theme when respondents answered all questions for the construct.

**TABLE 5:** Survey questions attributed to both the open communication and punitive concerns and the teamwork and staffing organizational constructs.

Open communication and punitive concerns	Wording	Scale
In this unit, we discuss ways to prevent errors from happening again	+	^*^
I'd be more likely to report errors/near misses if it were anonymous	–	^§^
Staff are afraid to ask questions when something does not seem right	–	^*^
My colleagues would report an error or near‐miss that they caused	+	^§^
Staff worry that mistakes they make are kept in their personnel file	–	^†^
My colleagues would report an error or near miss that I caused	+	^§^
Staff feel like their mistakes are held against them	–	^†^
Staff feel free to question decisions/actions of those with more authority	+	^*^
When an event is reported, it feels like the person is being written up, not the problem	–	^†^
Staff freely speak up if they see something that may negatively affect patient care	+	^*^
**Teamwork and Staffing**	Wording	Scale
We have enough staff to handle the workload	+	^†^
We use more agency/temporary staff than is best for patient care	–	^†^
When one area in this unit gets really busy, others help out	+	^†^
When a lot of work needs to get done quickly, we work together as a team	+	^†^
In this department, people treat each other with respect	+	^†^
People support one another in this department	+	^†^
We work in “crisis mode”, trying to do too much too quickly	–	^†^
When pressure builds up, my supervisor wants us to work faster even if it means taking shortcuts	–	^†^
Staff in this unit work longer hours than is best for patient care	–	^†^

* 5‐level Likert scale with options ranging from never (score = 1) to always (score = 5).

^†^ 5‐point Likert Scale with options ranging from strongly disagree (score = 1) to strongly agree (score = 5).

^§^ 6‐point Likert Scale with options ranging from I'd prefer not to answer (score = 1), strongly disagree (score = 2) to strongly agree (6).

*Note*: The scoring for all negatively worded questions were inverted prior to analysis.

Both organizational constructs demonstrated excellent internal reliability, with Cronbach's alpha coefficients of 0.82 and 0.89 for the teamwork and staffing and the open communication and punitive concern themes, respectively. Using Pearson product‐moment correlation, a small‐to‐moderate negative correlation was found between EE and the open communication and punitive concerns construct among therapy physicists (r(182) = −0.34, *p* < 0.001). A moderate‐to‐strong negative correlation was also found between EE and the teamwork and staffing construct among therapy physicists (r(217) = −0.61, p < 0.001).

We conducted linear regression analysis to examine the relationship between organizational and Likert scores for each of the three MBI domains, which indicates the frequency of experiencing each emotion. The analysis showed that for every 10‐point increase on the teamwork and staffing score, there was a 1.47‐point decrease in EE (*p* < 0.0001), a 0.69‐point decrease in DP (< 0.0001), and a 0.46‐point increase in PA (*p* < 0.0001). Similarly, for each 10‐point increase in the open communication and punitive concerns score, EE decreased by 0.72‐points (*p* < 0.0001), DP decreased by 0.42‐points (*p* < 0.0001), and PA increased by 0.42 points (*p* < 0.0001).

## 4 DISCUSSION

A total of 387 responses were received, resulting in a response rate of 20.1% relative to the number of deliverable invitations. Although this falls within the expected response rate for detailed online surveys,^22^ it is important to note that less than 2 weeks prior to the survey release, several U.S. hospitals were targeted in a ransomware attack. This led many institutions to increase their cybersecurity measures, including stricter spam filters and website access restrictions, which likely affected the survey's accessibility. Nearly 40% of invitations were never opened, highlighting the challenges of external factors on survey participation.

The external validity of the results was assessed by comparing the demographics of respondents to those of other data sources. For example, 36.2% of respondents identified as working in an academic environment, which is similar to the 32.1% reported in the AAPM TG‐275.S data.^23^ Additionally, 72.3% of participants reported working as therapy physicists compared to 76% of respondents who reported working as therapy physicists in the AAPM salary survey conducted during the same calendar year.^24^ The distribution of respondents also captured a broad range of clinic sizes. The good agreement between the study's respondent demographics and other data sources suggests that the findings are representative of the broader medical physics community in the United States.

**FIGURE 5:**
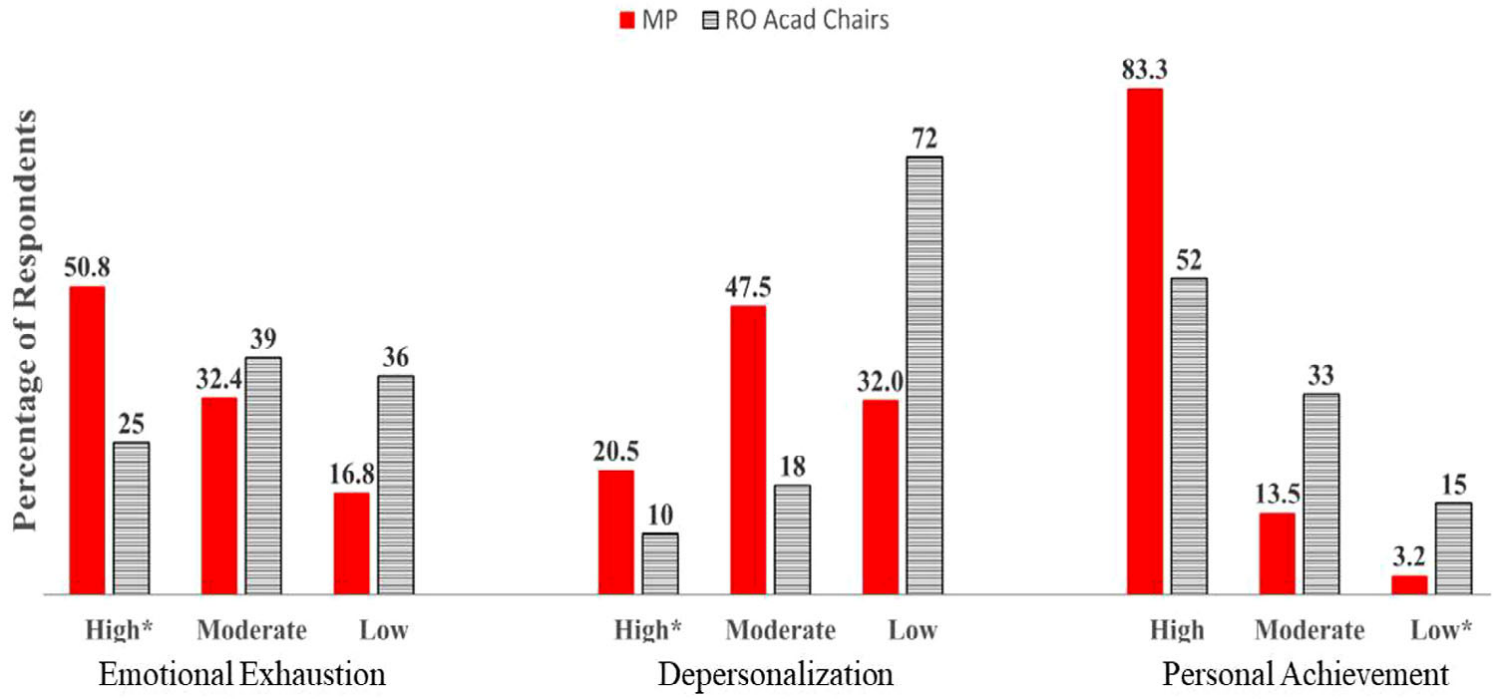
Burnout risk among medical physicists compared with the published burnout results of radiation oncology academic chairs.^23^ Both studies used the MBI and the historical cut score methodology. * Indicates high‐risk category on each domain. MBI, Maslach Burnout Inventory.

Using the historic cut score methodology, the percentage of medical physicists experiencing burnout can be compared with medical professionals in related fields. Using pooled MBI data from five studies, a meta‐analysis found that 38.7%, 21.5%, and 28% of radiation therapists experienced high EE, high DP, and low PA respectively.^25^ In comparison, medical physicists experience higher levels of EE (50.8%), similar levels of DP (20.5%), but much lower levels of reduced PA (3.2%) than radiation therapists. A study on burnout in interventional radiologists^26^, identified that 61.9%, 54.3% and 14.7% were experiencing high EE, high DP, and low PA respectively, which was worse across all three domains compared to medical physicists. Another comparison (Figure 5) illustrates that the proportion of medical physicists at high risk for EE and DP was double that reported in a study on burnout in academic chairs of radiation oncology departments.^27^ Notably, 68% of medical physicist respondents fall into the high and moderate risk categories on the DP scale compared to just 28% of the academic chairs.

Although the current preferred scoring method shows a lower percentage of medical physicists classified as high‐risk for burnout, the frequency with which these feelings occur is troubling. Specifically, 12% of respondents fell into the DP high‐risk category and experience the associated emotions almost weekly, while the overall respondent group experienced the emotion less than once per month. Given that quality and safety are central to the work of medical physicists, a lack of concern or caring for the individuals served is particularly alarming. There is also concern for the overall well‐being of the medical physicist experiencing these emotions.

Although only 6.6% of respondents can be classified as experiencing full burnout, indicated by high‐risk classification across all three dimensions, another 53.3% are at risk of developing full burnout without intervention. Interestingly, it is high levels of PA experienced by medical physicists that may be providing a protective buffer against burnout. Although high levels of PA were consistent regardless of the practiced sub‐specialty, therapy physicists demonstrated higher levels of EE and DP than their non‐therapy colleagues. This disparity may be attributed to the populations served by each. Therapy physicists primarily provide treatment to cancer patients with radiation doses that are orders of magnitude larger than those used in other sub‐specialties. The potential consequences of errors within the therapy specialty, highlighted in media reports over the past two decades, may contribute to heightened levels of burnout risk. Further, while therapy physicists are primarily focused on treating patients with a malignancy, diagnostic imaging, for example, is used for a variety of purposes including routine screenings.

Most respondents reported working between 40–59 h per week. However, 11% reported working between 60 and 99 h per week. PA was unaffected by the average number of hours worked per week. We speculate that this result may stem from feelings of making a positive impact on the patients served or because of increased financial compensation. Although PA was unaffected, the risk of EE and DP rose with increasing work hours. This is unsurprising since extended hours may suggest a low resource environment, such as an understaffed workplace. Burnout risk has also been demonstrated to increase when long work hours are chronic, particularly when there is insufficient time for recovery between very active periods.^28^

A significant inverse relationship was found between all three burnout domains and the department safety score provided by respondents. This research cannot identify whether higher burnout scores negatively affect staff perceptions of the workplace or if an inferior department safety profile is contributing to staff burnout risk. However, research involving data from 164 hospitals and over 140,000 providers and staff indicated that composite scores from the Agency for Healthcare Research and Quality Surveys on Patient Safety (version 1), the instrument upon which the organizational survey component of this research is based, was positively correlated with safety scores from multiple, independent consumer reporting sources.^29^ This suggests that staff perceptions of safety are consistent with objective assessments from independent sources and may potentially serve as an indicator of burnout risk. The results also highlight the impact and role of a sub‐optimal work environment on individual burnout risk.

COVID‐19 has had a profound impact on many healthcare workers. One meta‐analysis evaluated the psychological impact of the pandemic, including anxiety, depression, stress, and burnout, on healthcare workers.^30^ The pooled prevalence of burnout from three articles (*n* = 2,487) was 37.4%. Although most participants in the current study indicated that the pandemic had little to no impact on their job‐related feelings, 35.3% of participants reported that it had a moderate or significant impact on them. Further, this latter group experienced significantly higher EE and DP compared to their peers. Although the driving factors for this relationship between Covid‐19 and burnout risk among medical physicists is unknown, another publication of healthcare workers in northern Italy suggests that workload/work hours, fear of acquiring the virus, and contact with COVID‐19 positive patients increased EE and DP while the ability to work remotely decreased EE and DP.^31^ Medical physicist respondents who were more significantly affected by the pandemic may have had less work flexibility, such as a remote or hybrid work option, or may have worked in an environment with inadequate supplies of personal protective equipment increasing transmission fears. Interestingly, no significant correlation between COVID‐19 and PA was observed in this study.

**FIGURE 6:**
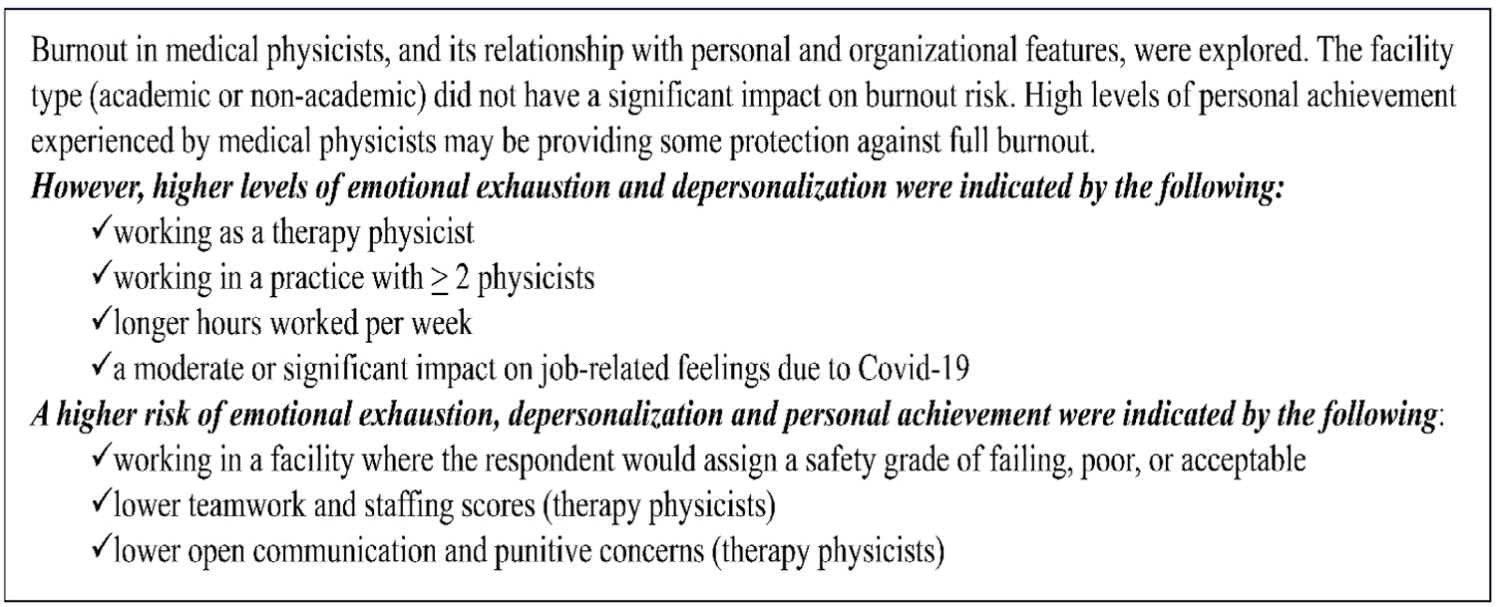
A summary of the relationships between burnout risk and job features among medical physicists in the United States.

There was a significant inverse relationship between EE and both the teamwork and staffing, and open communication and punitive concerns constructs among therapy medical physicists. This can be explained by the multidimensional theory of burnout, which suggests that employees value six key areas of work life, including workload, control or autonomy, reward, community, fairness, and values.^32^ A greater mismatch between an individual's values and those of their employer in these six areas can increase the risk of burnout. In this study, the workload and community components of the multidimensional theory are captured in the teamwork and staffing construct, while fairness, reward, and values are captured in the open communication and punitive concerns construct. The survey results underscore the significant influence of the work environment on staff burnout risk. Given the potential negative effects of burnout, it would be prudent for individuals to evaluate their own values in these six areas and consider how well current and prospective employers align with those values to mitigate burnout risk. Figure 6 provides a summary of the established relationships between burnout risk and both personal and organizational features identified in this study.

The current study has several limitations, including the potential bias of sampling only “healthy workers” who have remained in the field. Approximately three‐fourths (74.7%) of respondents had 21 or more years of experience, and 95.2% had spent more than half of their adult life in the United States. As a result, the generalizability of the findings to individuals with less experience or those who lived a shorter time in the US isn't clear. Although associations between burnout domains and work characteristics were identified, causation cannot be inferred. Additionally, as a cross‐sectional study, the data offers a snapshot in time and does not provide information on the longitudinal impact.

## 5 CONCLUSIONS

This is the first study to utilize a validated instrument to assess the prevalence of burnout among medical physicists in the United States and to examine the relationship between burnout and organizational features. The findings reveal that medical physicists are at high risk for burnout, with burnout levels exceeding those found in chairs of radiation oncology departments. Although only 6.6% of participants can be classified as experiencing full burnout using the new critical boundary scoring methodology, another 53.3% are demonstrating burnout tendencies and may be at risk for progressing to full burnout without intervention. This study also found a statistically significant relationship between burnout scores and various work environment factors. Higher facility safety scores, as well as better teamwork and staffing, and open communication (with fewer punitive concerns), were associated with lower burnout risk.

## AUTHOR CONTRIBUTIONS

Authors Deborah Schofield, C. Lynn Chevalier, Laurence Court, and Akiva Turner contributed to the study design. Authors Deborah Schofield and William Harmsen contributed to the statistical analysis. Author Deborah Schofield wrote the first draft of the manuscript. All authors agreed upon the final submitted manuscript.

## CONFLICT OF INTEREST STATEMENT

The authors declare no conflicts of interest.
